# ITHUIM: An efficient algorithm for mining inter-transaction high utility itemsets

**DOI:** 10.1371/journal.pone.0361061

**Published:** 2026-10-30

**Authors:** Vinh V. Vu, Thiet T. Pham, Bay Vo

**Affiliations:** 1 Faculty of Information Technology, Ho Chi Minh City University of Industry and Trade, Ho Chi Minh City, Vietnam; 2 Faculty of Information Technology, Industrial University of Ho Chi Minh City, Ho Chi Minh City, Viet Nam; 3 Faculty of Information Technology, HUTECH University, Ho Chi Minh City, Viet Nam; Lucian Blaga University of Sibiu: Universitatea Lucian Blaga din Sibiu, ROMANIA

## Abstract

Data mining is an active and vital research field that has led to the development of numerous algorithms and solutions. One significant contribution of this field is the discovery of high utility itemsets (HUIs) in transactional databases, which provides managers with valuable insights for maximizing profit. However, existing work on HUI mining has focused exclusively on the relationship among items within individual transactions, while ignoring relationships that occur across consecutive transactions. To address this limitation, we extend high utility itemset mining by considering mega-transaction relationships. We introduce the concept of mega-transaction and provide related definitions, formally defining the problem of mining inter-transaction high utility itemsets (ITHUIs). Furthermore, we develop and prove several mathematical properties that form the theoretical foundation for practical ITHUI mining. Based on these properties, a candidate pruning technique exploiting extended items is proposed to reduce the search space. Finally, we design a novel mining algorithm for efficiently discovering ITHUIs, named ITHUIM (ITHUI Miner).

## Introduction

Since the 1990s, extensive research has been devoted to frequent itemset mining (FIM) and association rule mining (ARM). Although these studies have produced numerous algorithms, they primarily rely on itemset frequency and do not consider other important factors, such as the profit, weight, or interestingness of items.

High utility itemset mining (HUIM) extends FIM by incorporating both the quantity and profit of items when assessing their importance. HUIM aims to discover itemsets whose utility satisfies a user-defined minimum utility threshold, denoted as *minUtil*. Many algorithms have been designed for HUIM. Early two-phase algorithms, such as Two-Phase [1], UP-Growth [2], and UP-Growth+ [3], first identify candidates with a high TWU (Transaction Weighted Utilization) and then compute the exact utility of each candidate in a second phase to obtain HUIs. However, two-phase methods tend to generate a massive number of candidates, even though only a few itemsets may have high utility, which may lead to long runtime and high memory usage.

To improve HUIM efficiency, several one-phase algorithms have been developed. HUI-Miner [4] introduced the utility-list structure to directly mine HUIs without generating candidates. Subsequently, EFIM [5] proposed two highly optimized techniques, High-utility Database Projection (HDP) and High-utility Transaction Merging (HTM), significantly improve performance. HMiner [6] also contributed a Compact Utility List (CUL) structure, along with several powerful pruning strategies. HUIM remains an active research topic, with ongoing studies focusing on global optimization, clustering-based mining, and parallel computation.

Despite this progress, traditional FIM and HUIM consider only relationships among items within a transaction. They do not capture patterns that span consecutive transactions and, therefore, cannot identify rules involving such temporal dependencies. For example, consider the two following rules:

Rule **L1**: *“If a customer buys product A, there will also be customers who purchase products B and C.”*

Rule **L2**: *“If a customer purchases product A, product B can be sold within three days, and product C will be sold in approximately five days.”*

Although traditional FIM and HUIM approaches can generate rules such as L1, they cannot produce rules such as L2. To address such temporal relationships, research on inter-transaction pattern mining emerged. Since then, many studies have been published to solve the problem of mining frequent inter-transaction itemsets. In 2000, Lu et al. [7] proposed the E-Apriori algorithm, and Bastide et al. [8] proposed the Eh-Apriori algorithm to extract inter-transaction patterns (ITPs) using the Apriori algorithm. In 2002, Feng et al. [9] enhanced the EH-Apriori algorithm for mining inter-transactional association rules under rule templates by using joining, converging, and speeding techniques. Subsequently, the FITI algorithm [10] was introduced in 2003. In 2007, Lee and Wang proposed ITP-Miner [11] to more efficiently mine ITPs in terms of time and memory usage. In 2017 and 2019, Nguyen et al. developed two algorithms, DITP-Miner [12] and FCITP-Miner [13], to mine closed inter-transaction patterns. In 2020, Nguyen et al. introduced the NL-ITP-Miner algorithm [14], which applies the N-list structure to inter-transaction pattern mining.

However, the method for mining frequent inter-transaction itemsets cannot be directly applied to the high utility setting due to fundamental differences between frequency and utility. Therefore, in this study, we focus on the problem of discovering inter-transaction high utility itemsets (ITHUIs). The main contributions of this paper are as follows:

- We formally define the problem of mining inter-transaction high utility itemsets.
- We provide rigorous mathematical formulations and proofs that establish the theoretical foundation for ITHUI mining.
- We present a theorem that describes the relationship between the sum of utility and the remaining utility for the extension-items.
- We propose ITHUIM (ITHUI Miner), a novel algorithm inspired by iEFIM designed to efficiently mine ITHUIs.
- We introduce fast upper-bound calculation techniques for extension-items.
- We design an effective candidate pruning strategy to reduce the search space during the mining process.

The remainder of this paper is organized as follows. The Related Work section reviews previous studies on high utility itemset mining, and inter-transaction itemset mining. The Basic Concepts and Problem Statement section introduces the key definitions and properties and formally defines the problems of mining high utility itemsets from inter-transaction databases. The Proposed Solution section presents the proposed approach for efficiently mining HUIs from inter-transaction databases. The Experimental Results section presents and analyzes the experimental results to evaluate the performance of the proposed approach. Finally, the Conclusion and Future Work section summarizes the main findings and outlines directions for future research.

## Related work

### High utility itemset mining

HUIM was introduced to address an essential limitation of FIM. Traditional FIM algorithms only consider whether an item appears in a transaction (binary presence), implicitly assuming all items contribute equally. However,in many real applications, especially in retail and commerce, items differ substantially in terms of unit profit, importance, or quantity sold. For example, selling one refrigerator yields far more profit than selling a needle or a loaf of bread, and selling products in larger quantities generates higher revenue. HUIM incorporates both item quantities and unit profits to identify itemsets that generate high overall utility.

Due to its practical relevance, HUIM has garnered considerable research attention, resulting in a diverse range of algorithms for real-life applications. Existing HUIM algorithms can be broadly classified into two-phase and one-phase approaches. Two-phase algorithms operate in two steps. In first step, they use an upper bound to reduce the search space and generate candidates that satisfy the upper bound value. In second step, they accurately calculate the utility value of each candidate to identify the actual HUIs. In contrast, one-phase algorithms generate candidate and compute their utility values simultaneously during the mining process by exploiting compact data structures and powerful pruning strategies, thereby reducing both execution time and memory consumption.

A significant milestone in HUIM research is the one-phase HUI-Miner algorithm, proposed by Liu and Qu [4] in 2012. HUI-Miner introduced the Utility List (UL) structure, which compactly stores the exact utility and remaining utility of itemsets. It relies on UL structure, a TWU upper bound for reducing the search space, and a pruning strategy based on the sum of utility and remaining utility to eliminate unpromising candidates. However, HUI-Miner requires constructing numerous intermediate utility lists during candidate generation. This process becomes computationally costly, especially on sparse datasets where many candidates are not HUIs. In 2014, the FHM algorithm [15] was introduced to address this limitation. Instead of simply generating all candidates, FHM utilizes a data structure called the Estimated Utility Co-occurrence Structure (EUCS) to store the TWU values of 2-itemsets. The EUCS efficiently identifies whether a pair of elements {*x*, *y*} is a promising candidate, thereby pruning the search space early. Experiments have demonstrated that the FHM algorithm is significantly more effective than HUI-Miner, particularly in handling sparse data.

In 2017, Zida et al. proposed EFIM [5], which remains one of the most efficient HUIM algorithms to date. EFIM introduced two practical upper bounds, local utility (lu) and subtree utility (su), along with two techniques namely high utility database projection and high utility transaction merging. These techniques effectively eliminate candidates and reduce the cost of database scans for calculating utility values. EFIM also employed an array-based approach to quickly calculate and update the lu and su values of itemsets, thereby optimizing memory usage. Subsequently, Krishnamoorthy proposed the concept of closed and non-closed transactions for an itemset prefix X. Based on these ideas, the Compact Utility List (CUL) structure was developed, enabling the HMiner algorithm [6] to achieve robust performance on both dense and sparse datasets. In 2019, Nguyen et al. [16] proposed iEFIM, an improved version of EFIM. iEFIM introduced the P-set structure that stores only the exact transactions containing a candidate itemset, which significantly reduces the overhead of projected databases required. In 2021, Wang et al. proposed two efficient HUIM algorithms called HUIL-TN and HUI-TN [17]. The key of these two algorithms is based on a Tail-Node tree structure to directly retrieve the exact utility values of itemsets without rescanning the database or creating candidates.

Several recent works have explored alternative search structures or incorporated parallelization. Qu et al. developed a one-phase algorithm called Hamm [18] by combining the benefits of Prefix Trees and Utility Vectors. Moreover, they proposed an optimization method applied within Hamm to reduce the search space and increase the algorithm’s execution speed. In 2023, Kumar and Mohbey [19] developed the Communication Cost Effective Utility-based Pattern Mining (CEUPM) algorithm using the Spark framework. The algorithm partitions the search space of HUIM for parallel processing, resulting in improved performance. The researchers relied on calculating the number of candidates that needed to be processed to determine the algorithm’s performance. Additionally, they proposed a method to optimize communication costs, further enhancing the algorithm’s efficiency. Fang et al. [20] proposed PHA-HUIM, a hybrid parallel and heuristic algorithm that leverages GPU computation. It integrates a novel data structure combined with a sort-mapping strategy and a multi-start strategy to accelerate the search for HUIs. PHA-HUIM performs three phases: search, fitness evaluation, and ring topology communication. To eliminate the costs of generating new candidates, Yan et al. [21] proposed an efficient vertical algorithm, namely FOTH (Fast sOrted iTemset searcH), using the data structure IndexSet. In 2025, Yang et al. [22] introduced a new method for HUIM that utilizes particle swarm optimization to enhance the Lévy flight mechanism in cuckoo search.

In practice, individual products are generalized into product categories and organized into hierarchical structures. For example, products like “Coca-Cola” and “Pepsi” are classified as “soft drinks,” while “pear” and “apple” are classified as “fruits.” In the high utility set mining problem, the focus is on specific items, such as “Pepsi,” “Coca-Cola,” “pear,” and “apple,” while ignoring more general items like “soft drinks” or “fruits.” In 2017, Cagliero et al. [23] were the first to propose the high utility set mining problem based on this hierarchical database structure. The problem is divided into two main types: mining sets containing items at the same level of the hierarchical tree (Multi-level HUIM – MLHUIM) and mining high utility sets at different levels with a descendant relationship (Cross-level HUIM – CLHUIM).

In 2017, the MLHUI-Miner algorithm operated at each level’s separate search space. Tung et al. [24] proposed the MLHMiner algorithm in 2022 based on extending HMiner in the mining process. To effectively mine MLHUI, Trinh et al. [25] applied a parallel processing model to the mining process with two algorithms MCML+ and MCML++. In 2025, Loan et al. [26] used many strategies and an upper-bound to speed up the mining of Top-K HUI on a hierarchical database called TK-MLHUI. To solve the CLHUIM problem, Fournier-Viger et al. [27] proposed the CLH-Miner algorithm in 2020 and applied strategies specific to mining items at different levels. In 2022, the FEACP algorithm [28] was introduced using a smaller upper bound to reduce the number of candidates. Tung et al. [29] combined item sorting and tighter upper bounds to prune the search space in the CLHUN algorithm.

### Frequent inter-transaction itemset and inter-sequence pattern mining

Traditional frequent and high utility itemset mining captures relationships among items within individual transactions, but ignores associations across consecutive transactions. This limitation led to the development of inter-transaction pattern mining, which aims to discover patterns spanning multiple transactions.

The first algorithms for mining frequent inter-transactions patterns (ITPs) were introduced by Lu et al. in 2000 [7]. Early Apriori-based approaches include E-Apiori [30], Eh-Apiori [9] and FITI [10]. Subsequent algorithms used projection techniques, the IT-Tree structure, and depth-first traversal approaches, such as ICMiner [30] and PITP-Miner [31]. Algorithms for mining closed inter-transactions frequent itemsets include FCITP-Miner [13] and DITP-Miner [12]. In 2020, Nguyen et al. proposed NL-ITP-Miner [14], an efficient algorithm for mining frequent inter-transaction itemsets based on the N-List structure. NL-ITP-Miner introduced a pruning strategy on 1-infrequent inter-transaction itemsets and techniques to calculate the support of inter-transaction patterns quickly.

Similar to the frequent inter-transaction pattern mining problem, the inter-transaction sequences mining problem attracted the interest of many researchers. In 2009, Wang and Lee [11] introduced the EISP-Miner algorithm to mine frequent inter-sequential patterns located in consecutive transactions in a database. EISP-Miner considered the items in consecutive transactions as an ordered sequence. In 2012, Vo et al. [32] applied the dynamic bit vector (DV) structure and proposed the DBV-ISP algorithm to identify ISPs more efficiently.

A more recent contribution by Nguyen et al. is the DBV-ISPMIC algorithm [33], which utilizes a well-structured search tree and DBV-PatternList structure to calculate the support of FISPs efficiently. But data duplication in these approaches increases runtime and memory usage. To address this, Anh Nguyen et al. introduced a new algorithm, called ISP-PI [34], to solve this problem by proposing the pseudo-IDList data structure and the ISP-IC pruning method, which reduces both pattern generation and memory usage.

Despite progress in frequent itemset and sequence mining, existing approaches fail to consider item utility values and quantities, focusing solely on item presence. This motivates our work on developing new concepts and algorithms for mining inter-transaction high utility itemsets, enabling the discovery of more informative patterns and high profit patterns.

As previously outlined in the introduction, traditional frequent itemset mining and high-utility itemset mining discover relationships only between items occurring within the same transaction. Although these items serve as the basis for constructing association rules, but they support only the generation of rules of type **L1** and cannot produce rules of type **L2**.

Inter-transaction high-utility itemset mining differs fundamentally from sequential pattern mining and timestamp-based temporal mining in three key aspects. First, in terms of representation, ITHUI treats span-annotated items within a mega-transaction as an unordered set, capturing their co-occurrence within a sliding window of width *maxSpan* without distinguishing which item occurs before or after another. In contrast, Sequential Pattern Mining (SPM) requires a strict ordering among events. For example, the pattern ⟨{a},{b}⟩ is entirely different from ⟨{b},{a}⟩, reflecting sequential behavioral relationships. Second, regarding the interestingness measure, ITHUI employs utility as its primary measure, a non-anti-monotone measure requiring specialized upper-bound pruning strategies, whereas traditional SPM relies on support, which satisfies the downward-closure property. Third, in terms of temporal representation, ITHUI represents time through discrete positional spans (0,1,...,maxSpan) derived from transaction ordering, making it applicable to conventional transaction databases where precise timestamps are unavailable or unnecessary. Timestamp-based temporal mining, by contrast, requires continuous absolute timestamps and models specific time gaps (e.g., “event *A* occurs within 3 days before event *B*”). Consequently, the overlap among mega-transactions in ITHUI reflects the evaluation of the same transaction under multiple contextual windows, analogous to *n*-gram models in natural language processing, where the same word appears in multiple *n*-grams to capture different local contexts, rather than artificially inflating item utilities.

## Basic concepts and problem statement

This section introduces key concepts, definitions, and the problem statement.

**Definition 1.** *Let* $I=\{x_{1},x_{2},x_{3},...,x_{m}\}$ *be a set of items, and* $T=\{t_{1},t_{2},t_{3},...,t_{n}\}$ *be a set of transaction identifiers. A transaction database D consists of a set of transactions where each transaction contains a transaction identifier and a list of items with their corresponding quantities.*

A transaction is represented as a pair $\langle tid,T_{tid}\rangle$ where tid∈T and $T_{tid}$ is an itemset with quantities. For an item $x_{i}\in T_{tid}$, its quantity (also called internal utility) is a positive number denoted as $IU(x_{i},T_{tid})$.

For example, Table 1 illustrates a transaction database *D* with 6 transactions, denoted as $D=\{\langle 1,T_{1}\rangle ,\langle 2,T_{2}\rangle ,\langle 3,T_{3}\rangle ,\langle 4,T_{4}\rangle ,\langle 5,T_{5}\rangle ,\langle 6,T_{6}\rangle \}$. In the first transaction *T*_1_, *IU*(*b*,*T*_1_) = 1, *IU*(*c*,*T*_1_) = 2. Furthermore, the transaction $\langle 6,T_{6}\rangle$ has *tid* = 6 and contains items *a*,*b*,*c*,*f* and quantities 3,4,1,2, respectively.

**Table 1:** Transaction dataset *D.*

Tid	Transaction
1	b(1), c(2), d(1), g(1)
2	a(4), b(1), c(3), d(1), e(2)
3	a(4), c(2), d(1)
4	c(2), e(1), f(1)
5	a(5), b(2), d(1), e(2)
6	a(3), b(4), c(1), f(2)

Each item $x_{i}\in I$ also has an external utility (profit), denoted as $EU(x_{i})$. For example, in Table 2, *EU*(*a*) = 1, *EU*(*b*) = 2, and *EU*(*d*) = 5.

**Table 2:** List of item profits in dataset *D.*

Item	a	b	c	d	e	f	g
**profit**	1	2	1	5	4	3	1

**Definition 2.** *An extension-transaction,* $T_{u}(\varphi )$*, derived from a base transaction* $\langle u,T_{u}\rangle =\{x_{1},x_{2},x_{3},...,x_{s}\}$ *with the internal utilities of the items being, respectively* $IU(x_{1},T_{u})=q_{x_{1}}$*,* $IU(x_{2},T_{u})=q_{x_{2}}$*, ...,* $IU(x_{s},T_{u})=q_{x_{s}}$*, and a given span value* φ≥0 *is defined as:*


$$\begin{array}{c} T_{u}(\varphi )=\{x_{1}(\varphi ,q_{x_{1}}),x_{2}(\varphi ,q_{x_{2}}),x_{3}(\varphi ,q_{x_{3}}),...,x_{s}(\varphi ,q_{x_{s}}\} \end{array}$$

**Key Concepts Explained:**

- $x_{i}(q_{x_{i}})$ is an item in the transaction $\langle u,T_{u}\rangle$ and $q_{x_{i}}=IU(x_{i},T_{u})$.
- φ is referred to as the (span) extension-span.
- $q_{x_{i}}=IU(x_{i}(\varphi ),T_{u}(\varphi ))=IU(x_{i},T_{u})$ is the internal utility of $x_{i}(\varphi )$ in the extension-transaction $T_{i}(\varphi )$
- $x_{i}(\varphi ,q_{x_{i}})$ is an extension-item of $x_{i}$ with *span* φ and $IU(x_{i}(\varphi ),T_{u}(\varphi ))=q_{x_{i}}$.

**Example.** Given a transaction $\langle 5,T_{5}\rangle$ and φ=4, we obtain:


$$\begin{array}{c} T_{u}(\varphi )=T_{5}(4)=\{a(4,5),b(4,2),d(4,1),e(4,2\}. \end{array}$$

**Definition 3.** *The utility of an extension-item* $x_{i}(\varphi )$ *in an extension-transaction* $T_{u}(\varphi )$*, denoted as* $u(x_{i}(\varphi ),T_{u}(\varphi ))$*, is defined as:*


$$\begin{array}{c} u(x_{i}(\varphi ),T_{u}(\varphi ))=IU(x_{i}(\varphi ),T_{u}(\varphi ))\times EU(x_{i}) \end{array}$$

**Example.** $u(a(4),T_{5}(4))=5\times EU(a)=5\times 1=5;u(e(4),T_{5}(4))=2\times EU(e)=2\times 4=8$.

**Definition 4.** *The transactional utility of* $T_{s}(\varphi )$ *is denoted by* $u(T_{s}(\varphi ))$ *and calculated as:*


$$\begin{array}{c} u(T_{s}(\varphi ))=\sum_{x_{i}(\varphi )\in T_{s}(\varphi )}u(x_{i}(\varphi ),T_{s}(\varphi )). \end{array}$$

**Example.**


$$\begin{array}{c} \begin{array}{cc} u(T_{5}(4)) & =u(a(4),T_{5}(4))+u(b(4),T_{5}(4))\\ & \;+u(d(4),T_{5}(4))+u(e(4),T_{5}(4))\\ & =5+4+5+8=22. \end{array} \end{array}$$

**Property 1.** *All extension-transactions of an original transaction have the same utility and equal transactional utility of the original transaction.*

*Proof.* With every φ and original transaction $\left(u,T_{u}\right)$ in *D* whereas


$$\begin{array}{c} T_{u}=\{x_{1}(q_{x_{1}}),x_{2}(q_{x_{2}}),x_{3}(q_{x_{3}}),...,x_{s}(q_{x_{s}}\},(\text{s is the cardinality of }T_{u}) \end{array}$$

We have:


$$\begin{array}{c} TU(T_{u})=\sum_{i=1}^{s}u(x_{i},T_{u})=\sum_{i=1}^{s}IU(x_{i},T_{u})\times EU(x_{i})=\sum_{i=1}^{s}q_{x_{i}}\times EU(x_{i}). \end{array}$$

$$\begin{array}{c} u(T_{u}(\varphi ))=\sum_{x_{i}(\varphi )T_{u}(\varphi )}u(x_{i}(\varphi ),T_{u}(\varphi ))=\sum_{x_{i}(\varphi )\in T_{u}(\varphi )}IU(x_{i}(\varphi ),T_{u}(\varphi ))\times EU(x_{i}) \end{array}$$

$$\begin{array}{c} =\sum_{x_{i}(\varphi )T_{u}(\varphi )}q_{x_{i}}\times EU(x_{i})=\sum_{i=1}^{s}q_{x_{i}}\times EU(x_{i}). \end{array}$$

Hence, ∀φ, $u(T_{u}(\varphi ))=TU(T_{u})$. □

**Definition 5.** *Given two extension-items* $x_{i}(\varphi_{i})$ *and* $x_{j}(\varphi_{j})$*:*


$$\begin{array}{c} x_{i}(\varphi_{i})<x_{j}(\varphi_{j})\;\text{if}[\begin{array}{cc} & \varphi_{i}<\varphi_{j},\\ & \varphi_{i}=\varphi_{j}\text{ and }x_{i}<x_{j}. \end{array} \end{array}$$

Moreover, $x_{i}(\varphi_{i})=x_{j}(\varphi_{j})$ if $\varphi_{i}=\varphi_{j}$ and $x_{i}=x_{j}$.

**Example.** *a*(0) < *b*(1), *a*(4) < *b*(4).

**Definition 6.** *An inter-transaction itemset is a set of extension-items:* $X=\{x_{1}(\varphi_{1}),x_{2}(\varphi_{2}),...,x_{k}(\varphi_{k}\}$*, where* $\varphi_{1}=0$*.* $\varphi_{k}<ℒ$*,* ℒ *is a user-defined maxSpan threshold,* $x_{i}(\varphi_{i})<x_{j}(\varphi_{j})$*, and* 1≤i≤j≤k*.*

**Definition 7.** *Given a transaction* $\langle \varphi_{1},T_{\varphi_{1}}\rangle$ *in the original transactional database D, a mega-transaction* $M_{\varphi_{1}}$ *is defined from D based on* $\varphi_{1}$*:*


$$\begin{array}{c} M_{\varphi_{1}}=T_{\varphi_{1}}(0)\cup T_{\varphi_{2}}(\varphi_{2}-\varphi_{1})\cup T_{\varphi_{3}}(\varphi_{3}-\varphi_{1})\cup \cdots \cup T_{\varphi_{k}}(\varphi_{k}-\varphi_{1}), \end{array}$$

*where* $\langle \varphi_{1},T_{\varphi_{1}}\rangle$, $\langle \varphi_{2},T_{\varphi_{2}}\rangle$*, ...,* $\langle \varphi_{k},T_{\varphi_{k}}\rangle$ *are consecutive transactions in the original transactional database D,* $\varphi_{i}<\varphi_{i+1}$*, i < k, and* $\varphi_{k}-\varphi_{1}<ℒ$.

Note that $M_{\varphi_{1}}$ is also an inter-transaction itemset, where $\varphi_{1}$ is the reference point.

In other words, with a given *maxSpan* value ℒ and transactional database *D*, a mega-transaction $M_{\varphi }$ is the union:


$$\begin{array}{c} M_{\varphi }={⋃}_{i=0}^{ℒ-1}T_{\varphi +i}(i)=T_{\varphi }(0)\cup T_{\varphi +1}(1)\cup \cdots \cup T_{\varphi +ℒ-1}(ℒ-1). \end{array}$$

**Definition 8.** *A mega-transaction database MegaD with maxSpan* ℒ *is the set of all mega-transactions created from D with* ℒ*.*

**Example.** Given the database *D* in Table 1, with ℒ=3, the mega-transaction database *MegaD* is defined in Table 3.

**Table 3:** Mega-transaction database *MegaD* generated from *D.*

Tid	Mega-transaction
1	b(0,1), c(0,2), d(0,1), g(0,1), a(1,4), b(1,1), c(1,3), d(1,1), e(1,2), a(2,4), c(2,2), d(2,1)
2	a(0,4), b(0,1), c(0,3), d(0,1), e(0,2), a(1,4), c(1,2), d(1,1), c(2,2), e(2,1), f(2,1)
3	a(0,4), c(0,2), d(0,1), c(1,2), e(1,1), f(1,1), a(2,5), b(2,2), d(2,1), e(2,2)
4	c(0,2), e(0,1), f(0,1), a(1,5), b(1,2), d(1,1), e(1,2), a(2,3), b(2,4), c(2,1), f(2,2)
5	a(0,5), b(0,2), d(0,1), e(0,2), a(1,3), b(1,4), c(1,1), f(1,2)
6	a(0,3), b(0,4), c(0,1), f(0,2)

Given the initial transaction database *D* shown in Table 1 and a *maxSpan* ℒ=3, each mega-transaction generated according to Definition 7 consists of a combination of up to *maxSpan* ℒ=3 consecutive transactions, starting with a selected reference transaction. Consequently, the mega-transaction with *Tid* = 1 is formed by concatenating the three extension-transactions $T_{1}(0)=\{b(0,1),c(0,2),d(0,1),g(0,1\},T_{2}(1)=\{a(1,4),b(1,1),c(1,3),d(1,1),e(1,2)\}$, and *T*_3_(2) = {*a*(2,4), *c*(2,2), *d*(2,1)} resulting in the mega-transaction shown in Table 3. The remaining mega-transactions are structured in a similar way

**Definition 9.** *Utility of an extension-item* $x_{i}(\varphi_{t})$ *in a mega-transaction* $M_{\varphi }$ *(*$x_{i}(\varphi_{t})\in M_{\varphi }$*) is denoted as* $u(x_{i}(\varphi_{t}),M_{\varphi })$ *and is computed as* $u(x_{i}(\varphi_{t}),M_{\varphi })=u(x_{i}(\varphi_{t}),T_{s}(\varphi_{t}))$*, where* $T_{s}(\varphi_{t})\subseteq M_{\varphi }\wedge x_{i}(\varphi_{t})\in T_{s}(\varphi_{t})$*.*

**Example.** $u(b(0),M_{1})=u(b(0),T_{1}(0))=1\times 2=2$, $u(b(1),M_{1})=2\times 1=2$.

**Definition 10.** *The utility of an inter-transaction itemset X in mega-transaction* $M_{\varphi }$ *is denoted as* $u(X,M_{\varphi })$ *and is defined as:*


$$\begin{array}{c} u(X,M_{\varphi })=\left\{ \begin{array}{cc} \sum_{x_{i}(\varphi_{t})\in X}u(x_{i}(\varphi_{t}),M_{\varphi }), & \text{if }X\subseteq M_{\varphi },\\ 0, & \text{otherwise}. \end{array}\right. \end{array}$$

**Example.** Given *X* = {*b*(0), *a*(1)}, we have *u*(*X*, *M*_1_) = 2 + 4 = 6, *u*(*X*, *M*_3_) = 0, *u*(*X*, *M*_5_) = 7.

**Definition 11.** *The utility of an inter-transaction itemset X in a mega-transaction database MegaD is denoted as u(X) and is defined as* $u(X)=\sum_{M_{\varphi }\in \text{MegaD}}u(X,M_{\varphi })$*.*

**Definition 12.** *An inter-transaction itemset* $X=\{x_{1}(\varphi_{1}),x_{2}(\varphi_{2}),...,x_{k}(\varphi_{k}\}$ *is an inter-transaction high utility itemset (ITHUI) if* u(X)≥minUtil*, otherwise X is an inter-transaction low utility itemset (ITLUI).*

**Problem Statement.** The problem of inter-transaction high utility itemset mining (ITHUIM) involves finding all itemsets in a mega-transaction database *MegaD* whose utility values are greater than or equal to a minimum utility threshold *minUtil*. In other words, the goal is to discover the set of ITHUIs: ITHUIs={X∣u(X)≥minUtil}.

**Example.** For the transaction database *D* in Table 1, with *maxSpan* ℒ=3 and *minUtil* = 50, the discovered ITHUIs are presented in Table 4.

**Table 4:** List of ITHUIs.

No	ITHUI	Utility of ITHUI
1	a(0), b(0), d(0), e(0), a(1), c(1)	51
2	a(0), d(0), e(0), a(1), c(1)	57
3	c(0), e(0), a(1), d(1), c(2), f(2)	52
4	b(0), c(0), d(0), a(1), c(1), d(1), c(2)	51
5	c(0), d(0), a(1), d(1), c(2)	52
6	c(0), d(0), c(1), e(1), a(2), d(2)	55
7	c(0), d(0), e(1), a(2), d(2)	50
8	d(0), c(1), e(1), a(2), d(2)	50
9	c(0), a(1), b(1), d(1), e(1), a(2)	52
10	c(0), a(1), b(1), d(1), e(1), a(2), c(2)	55

**Definition 13.** *The utility of a mega-transaction* $M_{\varphi }$*, denoted by* $MTU(M_{\varphi })$*, is defined as:* $MTU(M_{\varphi })=\sum_{T_{k}(i)\in M_{\varphi }}u(T_{k}(i))$*.* **Example.** For the running example, the MTU of all mega-transactions is provided in Table 5.

**Table 5:** The MTU of mega-transaction.

Mega-Transaction	*M*_1_	*M*_2_	*M*_3_	*M*_4_	*M*_5_	*M*_6_
**MTU**	43	42	42	49	40	18

**Property 2.** *Given an inter-transaction itemset X and a mega-transaction* $M_{\varphi }$ $\left(X\subseteq M_{\varphi }\right)$*, we have*


$$\begin{array}{c} u(X,M_{\varphi })\leq MTU(M_{\varphi }). \end{array}$$

*Proof.*


$$\begin{array}{c} MTU(M_{\varphi })=\sum_{T_{k}(i)\in M_{\varphi }}u(T_{k}(i))=\sum_{T_{k}(i)\in M_{\varphi }}\left(\sum_{x_{j}(i)\in T_{k}(i)}u(x_{j}(i),T_{k}(i))\right) \end{array}$$

$$\begin{array}{c} \geq \sum_{T_{k}(i)\in M_{\varphi }}\left(\sum_{\begin{array}{c} x_{j}(i)\in T_{k}(i)\\ x_{j}(i)\in X \end{array}}u(x_{j}(i),T_{k}(i))\right)=u(X,M_{\varphi }). \end{array}$$

Hence, $MTU(M_{\varphi })\geq u(X,M_{\varphi })$.□

**Property 3.** *Given a maxSpan* ℒ *and a transactional database D,*


$$\begin{array}{c} MTU(M_{\varphi })=MTU(M_{\varphi -1})-u(T_{\varphi -1}(0))+u(T_{\varphi +ℒ-1}(ℒ-1)) \end{array}$$

*Proof.*


$$\begin{array}{c} MTU(M_{\varphi })=\sum_{T_{k}(iinM_{\varphi }}u(T_{k}(i))=\sum_{i=0}^{i<ℒ}u(T_{\varphi +i}(i)) \end{array}$$

$$\begin{array}{c} =u(T_{\varphi }(0))+u(T_{\varphi +1}(1))+\cdots +u(T_{\varphi +ℒ-1}(ℒ-1)). \end{array}$$

$$\begin{array}{c} MTU(M_{\varphi -1})=\sum_{T_{k}(iinM_{\varphi -1}}u(T_{k}(i))=\sum_{i=0}^{i<ℒ}u(T_{\varphi -1+i}(i)) \end{array}$$

$$\begin{array}{c} =u(T_{\varphi -1}(0))+u(T_{\varphi }(1))+\cdots +u(T_{\varphi +ℒ-2}(ℒ-1)). \end{array}$$

Hence,


$$\begin{array}{c} \begin{array}{cc} MTU(M_{\varphi })-MTU(M_{\varphi -1}) & =\sum_{i=0}^{i<ℒ}u(T_{\varphi +i}(i))-\sum_{i=0}^{i<ℒ}u(T_{\varphi -1+i}(i))\\ & =u(T_{\varphi }(0))+u(T_{\varphi +1}(1))+\cdots +u(T_{\varphi +ℒ-1}(ℒ-1)\\ & -\left(u(T_{\varphi -1}(0))+u(T_{\varphi }(1))+\cdots +u(T_{\varphi +ℒ-2}(ℒ-1)\right)\\ & =\left(u(T_{\varphi }(0))-u(T_{\varphi }(1))\right)+\left(u(T_{\varphi +1}(1))-u(T_{\varphi +1}(2))\right)+\cdots \\ & +\left(u(T_{\varphi +ℒ-2}(ℒ-2))-u(T_{\varphi +ℒ-2}(ℒ-1))\right)\\ & +u(T_{\varphi +ℒ-1}(ℒ-1))-u(T_{\varphi -1}(0)). \end{array} \end{array}$$

As Property 1: $\forall \varphi ,\;u(T_{u}(\varphi ))=TU(T_{u})$, we have:


$$\begin{array}{c} \forall x,y:u(T_{u}(x))-u(T_{u}(y))=TU(T_{u})-TU(T_{u})=0. \end{array}$$

Therefore,


$$\begin{array}{c} MTU(M_{\varphi })-MTU(M_{\varphi -1})=u(T_{\varphi +ℒ-1}(ℒ-1))-u(T_{\varphi -1}(0)). \end{array}$$

or,


$$\begin{array}{c} MTU(M_{\varphi })=MTU(M_{\varphi -1})-u(T_{\varphi -1}(0))+u(T_{\varphi +ℒ-1}(ℒ-1)). \end{array}$$

**Definition 14.** *Mega-transaction Weight Utilization of inter-transaction itemset X is denoted as MTWU(X) and is computed as* $MTWU(X)=\sum_{X\subseteq M_{\varphi }}MTU(M_{\varphi })$*.*

**Example.** For the running example, the MTWU of inter-transaction 1-itemset *X* are provided in Table 6.

**Table 6:** MTWU of extension-items.

X Span	a	b	c	d	e	f	g
0	142	143	194	167	131	67	43
1	174	132	167	134	134	82	0
2	134	91	134	85	84	91	0

**Property 4.** *Given an inter-transaction itemset X, the following property holds:*


$$\begin{array}{c} \forall Y⊇X,\;MTWU(Y)\leq MTWU(X). \end{array}$$

*Proof.*


$$\begin{array}{c} \begin{array}{cc} MTWU(X) & =\sum_{\begin{array}{c} X\subseteq M_{\varphi } \end{array}}MTU(M_{\varphi })\\ & =\sum_{\begin{array}{c} X\subseteq M_{\varphi }\\ Y\subseteq M_{\varphi } \end{array}}MTU(M_{\varphi })+\sum_{\begin{array}{c} X\subseteq M_{\varphi }\\ Y⊈M_{\varphi } \end{array}}MTU(M_{\varphi })\\ & =\sum_{\begin{array}{c} Y\subseteq M_{\varphi } \end{array}}MTU(M_{\varphi })+\sum_{\begin{array}{c} X\subseteq M_{\varphi }\\ Y\subseteq M_{\varphi } \end{array}}MTU(M_{\varphi })\\ & =MTWU(Y)+\sum_{\begin{array}{c} X\subseteq M_{\varphi }\\ Y⊈M_{\varphi } \end{array}}MTU(M_{\varphi })\\ & \geq MTWU(Y) \end{array} \end{array}$$

(as $\sum_{\begin{array}{c} X\subseteq M_{\varphi }\\ Y⊈M_{\varphi } \end{array}}MTU(M_{\varphi })\geq 0$, we have: MTWU(X)≥MTWU(Y)).

So ∀Y⊇X, MTWU(X)≥MTWU(Y).□

**Property 5.** *The mega-transaction weight utilization of an inter-transaction itemset X is greater than or equal to the utility of X in megaD. That is* MTWU(X)≥u(X)*.*

*Proof.*


$$\begin{array}{c} \begin{array}{c} MTWU(X)=\sum_{\begin{array}{c} X\subseteq T_{\varphi }(i)\\ T_{\varphi }(iinM_{\varphi } \end{array}}MTU(M_{\varphi })=\sum_{\begin{array}{c} X\subseteq M_{\varphi }(i) \end{array}}MTU(M_{\varphi })\geq \sum_{\begin{array}{c} X\subseteq M_{\varphi } \end{array}}u(X,M_{\varphi })=u(X). \end{array} \end{array}$$

Thus, MTWU(X)≥u(X).□

**Definition 15.** *Given two inter-transaction itemsets* $X=\{x_{1}(\varphi_{1}),x_{2}(\varphi_{2}),...,x_{m}(\varphi_{m}\}$ *and* $Y=\{y_{1}(\omega_{1}),y_{2}(\omega_{2}),...,y_{m}(\omega_{n}\}$*.*

- *X* = *Y if* $x_{i}(\varphi_{i})=y_{i}(\omega_{i})$ *where* 1≤i≤m, $\varphi_{1}=\omega_{1}=0$ *and m* = *n*.
-
$X<Y\Leftrightarrow [\begin{array}{c} \exists k\in \{1,2,3,...,\min(m,n\}:\\ \;(\forall i<k:x_{i}(\varphi_{i})=y_{i}(\omega_{i})\;\wedge \;x_{k}(\varphi_{k})<y_{k}(\omega_{k})\\ \left(m<n)\;\wedge \;(\forall i\in \{1,2,3,...,\text{m}\}:x_{i}(\varphi_{i})=y_{i}(\omega_{i})\right) \end{array}$

**Property 6.** *Given an inter-transaction itemset X, if MTWU(X) <minUtil, then X and all its supersets are not ITHUIs.*

*Proof.* As in Property 5, MTWU(X)≥u(X), so if MTWU(X)<minUtil⇒u(X)<minUtil.

Moreover, ∀Y⊇X, MTWU(X)≥MTWU(Y) (as Property 4), thus ∀Y⊇X,


$$\begin{array}{c} u(Y)\leq MTWU(Y)\leq MTWU(X)<minUtil. \end{array}$$

Therefore, *X* and all supersets *Y* of *X* are not ITHUIs. □

**Definition 16.** *(Ordering of extension-items). The extension-items in the mega-transaction database are processed using a total order* ≻ *such that the extension-items are sorted in span ascending order. If extension-items have the same span, these extension-items are sorted in MTWU ascending order.*

**Example.** For the running example, the ordering of extension-items is g(0)≻f(0)≻e(0)≻a(0)≻b(0)≻d(0)≻c(0)≻g(1)≻f(1)≻b(1)≻d(1)≻e(1)≻c(1)≻a(1)≻g(2)≻e(2)≻d(2)≻b(2)≻f(2)≻a(2)≻c(2).

After all extension-items in the mega-transactions are updated according to the total order ≻, the resulting mega-transaction database is presented in Table 7.

**Table 7:** Mega-transaction database *MegaD* after updating.

Tid	Mega-Transaction
1	d(0,1), b(0,1), c(0,2), b(1,1), d(1,1), e(1,2), c(1,3), a(1,4), d(2,1), a(2,4), c(2,2)
2	e(0,2), a(0,4), d(0,1), b(0,1), c(0,3), d(1,1), c(1,2), a(1,4), e(2,1), f(2,1), c(2,2)
3	a(0,4), d(0,1), c(0,2), f(1,1), e(1,1), c(1,2), e(2,2), d(2,1), b(2,2), a(2,5)
4	f(0,1), e(0,1), c(0,2), b(1,2), d(1,1), e(1,2), a(1,5), b(2,4), f(2,2), a(2,3), c(2,1)
5	e(0,2), a(0,5), d(0,1), b(0,2), f(1,2), b(1,4), c(1,1), a(1,3)
6	f(0,2), a(0,3), b(0,4), c(0,1)

**Definition 17.** *Let* $M_{\varphi }⧵X$ *be the set of all extension-items after X in* $M_{\varphi }$


$$\begin{array}{c} M_{\varphi }⧵X=\left\{z(m)\in M_{\varphi }|\forall x(i)\in X:x(i)>z(m)\right\} \end{array}$$

**Example.** For the running example,


$$\begin{array}{c} X_{1}=\{e(0),b(0),c(1),a(1),e(2\},\;M_{2}⧵X_{1}=\{f(2),c(2)\} \end{array}$$

$$\begin{array}{c} X_{2}=\{e(0),c(1\},\;M_{2}⧵X_{2}=\{a(1),e(2),f(2),c(2)\},\;M_{5}⧵X_{2}=\{a(1)\}. \end{array}$$

**Definition 18.** *Given an inter-transaction itemset X, an extension-item z(m) is an extension of X if* ∀x(i)∈X,x(i)≻z(m)*, denoted as* X≻z(m)*. Let*


$$\begin{array}{c} Z(X)=\{\;z(m)|X≻z(m)\;\}. \end{array}$$

**Definition 19.** *Given an inter-transaction itemset X, the extension-itemsets of X are denoted by EX(X) and*


$$\begin{array}{c} EX(X)=\{\;Y=X\cup EZ|EZ\subseteq Z(X)\;\}. \end{array}$$

**Definition 20.** *The remaining utility of the inter-transaction itemset X in the mega-transaction database* $M_{\varphi }$ *is denoted as* $ru(X,M_{\varphi })$ *and calculated as the sum of the utilities of all extension-items after X according to the total order* ≻ *in* $M_{\varphi }$*:*


$$\begin{array}{c} ru(X,M_{\varphi })=\sum_{\begin{array}{c} x(i)\in M_{\varphi }⧵X \end{array}}u(x(i),M_{\varphi }). \end{array}$$

**Definition 21.** *The remaining utility of an inter-transaction itemset X in the mega-transaction database MegaD is denoted ru(X) and computed as*


$$\begin{array}{c} ru(X)=\sum_{\begin{array}{c} X\subseteq M_{\varphi }\\ M_{\varphi }\in MegaD \end{array}}ru(X,M_{\varphi }). \end{array}$$

**Property 7.** *Given an inter-transaction itemset X, if u(X) + ru(X) <minUtil, then X and EX(X) are ITLUIs.*

*Proof.* As *u*(*X*) + *ru*(*X*) <*minUtil* then *u*(*X*) <*minUtil*, so *X* is ITLUI.

Let $X^{\prime }\in EZ(X)$, then $X^{\prime }=X\cup EZ$ where EZ⊆Z(X).

- There exists $\forall M_{\varphi }\in MegaD$, $X^{\prime }\subseteq M_{\varphi }$:

$$\begin{array}{c} u(X^{\prime },M_{\varphi })=u(X,M_{\varphi })+u(RE,M_{\varphi }), \end{array}$$

- where $RE=X^{\prime }-X=\{x^{\prime }(t)|x^{\prime }(t)\in X^{\prime }\wedge x^{\prime }(t)\notin X\}$. It is easy to see that $RE\subseteq M_{\varphi }$ and $RE\subseteq M_{\varphi }⧵X$, thus $u(RE,M_{\varphi })\leq ru(X,M_{\varphi })$.
- Hence,

$$\begin{array}{c} u(X^{\prime },M_{\varphi })=u(X,M_{\varphi })+u(RE,M_{\varphi })\leq u(X,M_{\varphi })+ru(X,M_{\varphi }). \end{array}$$ (1)

- Let $\left\{MX=\{M_{\varphi }|M_{\varphi }\in MegaD\wedge X\subseteq M_{\varphi }\}\right\}$ and $\left\{MX^{\prime }=\{M_{\varphi }|M_{\varphi }\in MegaD\wedge X^{\prime }\subseteq M_{\varphi }\}\right\}$. As $X\subset X^{\prime }$ then $MX⊇MX^{\prime }$.
- Therefore,

$$\begin{array}{c} u(X)+ru(X)<minUtil \end{array}$$

$$\begin{array}{c} \Rightarrow \sum_{M_{\varphi }\in MX}u(X,M_{\varphi })+ru(X,M_{\varphi })<minUtil \end{array}$$

$$\begin{array}{c} \Rightarrow \sum_{M_{\varphi }\in MX^{\prime }}u(X,M_{\varphi })+ru(X,M_{\varphi })<minUtil \end{array}$$

$$\begin{array}{c} \Rightarrow \sum_{M_{\varphi }\in MX^{\prime }}u(X^{\prime },M_{\varphi })<minUtil\;\text{(from (1))} \end{array}$$

- Hence, $u(X^{\prime })<minUtil$, and $X^{\prime }$ is an ITLUI. □

To summarize the conceptual framework introduced above, we clarify the relationships among the key constructs. An extension transaction augments an original transaction with a span value that indicates its temporal offset relative to a reference time point, while a mega-transaction is formed by combining all extension transactions whose spans fall within the window. At the item level, an itemset is simply a set of original items, whereas an extension item is an original item annotated with a specific span value (e.g., *a*(0), *c*(2)). An inter-transaction itemset is then defined as a set of extension items that satisfies both the item-ordering constraint and the span constraint 0≤span<maxSpan. Given a user-specified minimum utility threshold *minUtil* and *maxSpan* ℒ, the problem addressed in this paper is to efficiently discover all inter-transaction high utility itemsets (ITHUIs).

## Proposed solution

### Baseline algorithm for mining inter-transaction high utility itemsets

In this section, we propose the ITHUIM algorithm to mine ITHUIs in a transaction database with a given *maxSpan* ℒ. This algorithm is developed based on the iEFIM algorithm with: $Pset(X)=\{\;M_{\varphi }.id|M_{\varphi }\in megaD\wedge X\subseteq M_{\varphi }\;\}$ is a set of identifiers of mega-transaction containing *X*; $Pex\_set(X,z)=\{\;M_{\varphi }.id|M_{\varphi }.id\in Pset(X)\wedge z\in M_{\varphi }\;\}$ is expansion of *Pset*(*X*) with item *z* on a mega-database.

**Algorithm 1: ITHUIM**

**Input:** transactional database *D*, *maxSpan* ℒ, user-threshold utility *minUtil*

**Output:** *ITHUIs*: a set of inter-transaction high utility itemsets

1: α=∅

2: Scan *D* and build a mega-transaction database *MegaD*

3: Calculate lu(α,i) values for all extension-items *i* in *MegaD*

4: Let Secondary(α) contain all extension-items having lu(α,i)≥minUtil

5: Revise all mega-transactions by using only extension-items in Secondary(α)

6: Build a total ascending order ≻ (as Definition 17)

7: Remove empty mega-transactions and sort extension-items in them by ≻

8: Scan *MegaD* to calculate su(α,i) and Pex_set(α,i) for each i∈Secondary(α)

9: Calculate Primary(α)={i∣i∈Secondary(α)∧su(α,i)≥minUtil}

10: ITHUIs=searchITHUI(α,MegaD,Primary(α),Secondary(α),minUtil,Pex_set(α,i))

For example, consider the transaction database *D* (Table 1), the profit list of items (Table 2), *minUtil* = 50 and ℒ=3. In this case, the ITHUI algorithm transforms *D* into a mega-transaction database *MegaD*, as shown in Table 3. Since *MegaD* is created from the original database *D*, the number of transactions in *MegaD* is the same as in *D*. Since each mega-transaction is created from multiple consecutive transactions, the number of extension-items in each mega-transaction is much greater than in the database *D*. To calculate the *MTWU* for all single extension-items, lu(α,i) with α=∅, the algorithm requires the determination of the *MTU* of all mega-transactions through the *TU* of transactions in *D* (Table 8). Instead of calculating the *MTU* value of mega-transactions independently, it is simpler and more effective to determine the MTUs by applying Property 3. The larger the value of ℒ, the more efficient this approach becomes.

**Table 8:** TU of original transactions in *D.*

Tid	1	2	3	4	5	6
**TU**	10	22	11	9	22	18

We have:


$$\begin{array}{c} \begin{array}{cc} MTU(M_{1}) & =TU(t_{1})+TU(t_{2})+TU(t_{3})=10+22+11=43\\ MTU(M_{2}) & =MTU(M_{1})+TU(t_{4})-TU(t_{1})=43+9-10=42\\ MTU(M_{3}) & =MTU(M_{2})+TU(t_{5})-TU(t_{2})=42+22-22=42\\ MTU(M_{4}) & =MTU(M_{3})+TU(t_{6})-TU(t_{3})=42+18-11=49 \end{array} \end{array}$$

The final MTU results are presented in Table 5.

The next step in processing the mega-transaction database using the proposed ITHUIM algorithm is to calculate all MTWU values, as presented in Table 6. After that, any extension-items having MTWU values lower than the minimum utility threshold (*minUtil*) are eliminated, and the remaining extension-items are sorted according to the total order ≻. Next, the ITHUIM algorithm rescans *MegaD* to calculate su(α,i) and Pex_set(α,i) for each item *i* in the secondary set. From these values, the algorithm determines Primary(α) and performs the mining of ITHUIs using the *searchITHUI* procedure based on a depth-first search strategy.

In *searchITHUI*, the algorithm sequentially combines α with each item *i* in the *Primary* set to generate extended candidates of α, denoted as β=α∪{i}. For each candidate β, the algorithm computes u(β) based on the list of transactions stored in Pset(α), while simultaneously constructing the projected database on β, denoted as $D_{\beta }$. If u(β) satisfies the minimum utility threshold, β is normalized and checked for duplication with any previously discovered ITHUI. If no duplication is found, β is added to the result set. Next, the algorithm computes lu(β,z), su(β,z), and Pex_set(β,z) for every extension-item *z* appearing after *i* according to the total order. Then, the sets Se(β) and Pri(β) are determined. Finally, the algorithm is recursively invoked to discover all ITHUIs in the projected database.

**Algorithm 2: searchITHUI**



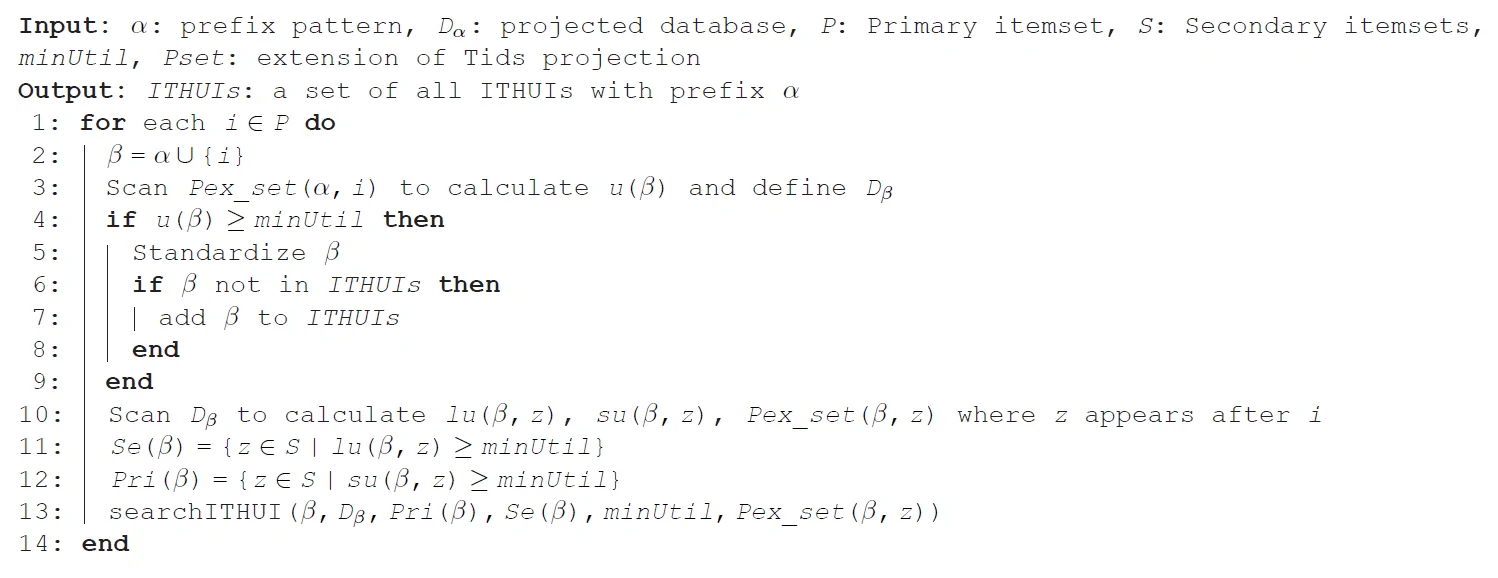



### Improving the efficiency of mining inter-transaction high utility itemsets

**Improvement 1**: *Expand only from single extension-items with span = 0*

During the process of ITHUI mining, when an itemset *X* is identified as a candidate whose utility value is equal to or greater than the specified minimum utility threshold (*minUtil*), the algorithm needs to normalize the itemset *X* and verify whether it already exists in the hash table. Based on this verification, the algorithm can determine whether *X* should be included in the set of discovered ITHUIs.

According to Definition 6, all itemsets must have an initial span equal to 0. Therefore, if an itemset does not satisfy this requirement, it must be standardized to ensure that its initial span becomes 0. To avoid generating candidates with an initial span different from 0, the algorithm only considers itemsets that are inevitably generated from extension-items having span = 0 during the exploration process.

**Theorem 1.** *For any inter-transaction itemset mined from the mega-transaction database whose initial span is not equal to 0, there exists an equivalent inter-transaction itemset that can be obtained with an initial span equal to 0.*

*Proof.* Suppose


$$\begin{array}{c} X=\{x_{0}(\varphi_{0}),x_{1}(\varphi_{1}),...,x_{k}(\varphi_{k}\} \end{array}$$

with u(X)≥minUtil. Let *Y* be the normalized itemset of *X*, defined as


$$\begin{array}{c} Y=\{x_{0}(\omega_{0}),x_{1}(\omega_{1}),...,x_{k}(\omega_{k}\}, \end{array}$$

where $\omega_{0}=0$ and $\omega_{i}=\varphi_{i}-\varphi_{0}$ for i≥1.

Assume that *MX* is the set of mega-transactions containing *X*. For any $M_{t}\in MX$, we have


$$\begin{array}{c} M_{t}=t_{a}(0)\cup t_{a+1}(1)\cup t_{a+2}(2)\cup \cdots \cup t_{a+\varphi_{0}}(\varphi_{0})\cup \cdots \cup t_{a+\varphi_{k}}(\varphi_{k})\cup \cdots . \end{array}$$

We can always locate a mega-transaction


$$\begin{array}{c} M_{t+\varphi_{0}}=t_{a+\varphi_{0}}(\varphi_{0}-\varphi_{0})\cup \cdots \cup t_{a+\varphi_{k}}(\varphi_{k}-\varphi_{0})\cup \cdots \end{array}$$

such that $Y\subseteq M_{t+\varphi_{0}}$.

According to Property 1, all extension-transactions derived from the same original transaction have identical utility. Therefore, for every $M_{t}\in MX$, there exists $M_{t+\varphi_{0}}\in MegaD$ such that


$$\begin{array}{c} u(X,M_{t})=u(Y,M_{t+\varphi_{0}}). \end{array}$$

Thus, we can always find an itemset *Y* normalized from *X* having the same utility value in the mega-transaction database. Therefore, in ITHUIM, the algorithm does not need to consider itemsets whose initial span is not equal to 0.□

**Algorithm 3: searchITHUI**



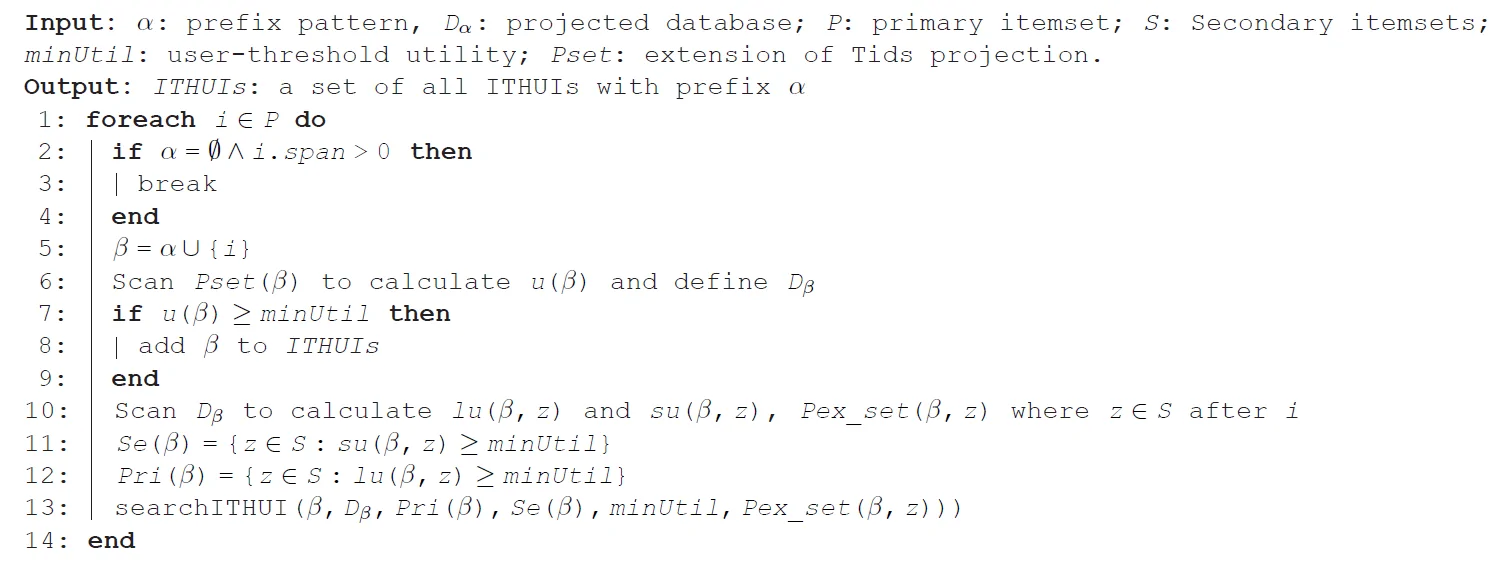



When the *searchITHUI* algorithm is first executed on α=∅, it only considers single extension-items with span equal to 0. This guarantees that no itemset having an initial span different from 0 is generated. Such a strategy reduces the number of generated candidates and minimizes the time required for candidate normalization and overlap verification.

Algorithm 2*a* is an improvement of the original Algorithm 2, in which items with span greater than 0 are not projected in order to eliminate duplicate candidates. Therefore, once each ITHUI is discovered, the algorithm does not require any normalization step and can directly add it to the final result set. This significantly reduces the number of candidates and narrows the search space. Moreover, the algorithm avoids additional normalization and verification phases before inserting patterns into the results.

**Improvement 2**: *Utilizing a new structure data to store extension-items, thereby decreasing memory storage and runtime*.

The information associated with each extension-item consists of two components: the item itself and its corresponding span. Therefore, to represent an extension-item, this study uses a pair ⟨int,byte⟩, where the integer value (*int*) stores the item identifier and the byte value (*byte*) stores the span. To further improve the execution speed and memory efficiency of the algorithm, this study proposes two methods for storing extension-items, as described below:

**Method 1**: Instead of using two separate variables, this study employs a single integer variable to encode both the item and its span. The most significant bit (the first bit) is unused because it represents the sign bit of the integer. The following 7 bits (bits 2–8) are used to store the span value, while the remaining 24 bits are used to store the item identifier. With 7 bits allocated for the span, the maximum representable span value is 127. Similarly, using 24 bits for the item allows storing item identifiers up to 2^24^.

**Table:** 

1	2	3	4	5	6	7	8	9	10	⋯	31	32
Sign	span							item				

**Method 2:** The study consolidates each extension-item into a single integer value. First, the algorithm determines the largest item in the original database, denoted as MAX_ITEM. Then, each extension-item is transformed into an integer according to the following formula:


$$\begin{array}{c} \text{newItem}=item+MAX\_ITEM\times span. \end{array}$$

**Improvement 3**: *Mining inter-transaction high utility itemsets without building a mega-transaction database*.

This study introduces an optimization that eliminates the need to explicitly construct a mega-transaction database. Recall that a mega-transaction database has the same number of transactions as the original database, but each mega-transaction may contain up to ℒ times more items due to span extensions. Consequently, building such a database requires substantial memory consumption and increases the scanning time during the mining process. To address this issue, the proposed approach directly mines ITHUIs from the original transaction database without materializing the mega-transactions.

The main challenge of this approach lies in computing the MTWU values of single extension-items, establishing the item ordering, constructing the EUCS structure, and generating the initial utility lists (1-ITHULs) without relying on MegaD.

**Calculating MTWU of single extension-items.** To compute the MTWU values, the algorithm first calculates the TU values (Table 8) and the MTU values (Table 5) of all transactions and corresponding mega-transactions based on Property 3. After that, the algorithm scans each transaction $\langle u,T_{u}\rangle$ in the original database *D*. For each transaction, the algorithm determines the maximum span that can be generated, denoted as *max*, which is computed as:


$$\begin{array}{c} max=(u\geq ℒ)?(ℒ-1):(u-1). \end{array}$$

For each item *x* in $\langle u,T_{u}\rangle$, the algorithm updates the MTWU values of all possible extension-items x(0),x(1),...,x(max) using the corresponding MTU values $MTU(M_{u}),MTU(M_{u-1}),...,MTU(M_{u-ℒ+1})$. This process continues until all transactions in *D* have been processed.

For example, given the database *D* with *maxSpan* ℒ=3 in Table 1, after determining the TU and MTU values, the algorithm initializes the MTWU values of all extension-items to zero before performing the updates in Table 9.

**Table 9:** Initialize MTWU equals zero for all extension-items.

X Span	*a*	*b*	*c*	*d*	*e*	*f*	*g*
0	0	0	0	0	0	0	0
1	0	0	0	0	0	0	0
2	0	0	0	0	0	0	0

This optimization significantly reduces both memory consumption and database traversal cost, while preserving the correctness of the mining results. As a consequence, the proposed method improves the scalability of the algorithm when handling large datasets or large span constraints.

In Step 1 of the algorithm, the process starts with the transaction $\langle u,T_{u}\rangle =\langle 1,T_{1}\rangle$ consisting of items {*b*(1), *c*(2), *d*(1), *g*(1)}. Since *u* = 1 and *max* = 0, the items only generate extension-items with span = 0. The al*g*orithm then examines each item in *T*_1_ sequentially and updates the corresponding MTWU values. Specifically, the values to be updated are *b*(0), *c*(0), *d*(0), and *g*(0), each increased by *MTU*(*M*_1_) = 43. The resulting MTWU table is shown in Table 10.

**Table 10:** MTWU values after scanning $\langle 1,T_{1}\rangle$.

X Span	*a*	*b*	*c*	*d*	*e*	*f*	*g*
0	0	43	43	43	0	0	43
1	0	0	0	0	0	0	0
2	0	0	0	0	0	0	0

In Step 2, the algorithm processes the transaction $\langle u,T_{u}\rangle =\langle 2,T_{2}\rangle$ with *T*_2_ = {*a*(4), *b*(1), *c*(3), *d*(1), *e*(2)}. Since *u* = 2, we obtain *max* = 1. Therefore, the items in *T*_2_ generate extension-items with span values of both 0 and 1. For each item in *T*_2_, the MTWU values are updated accordingly. The updates include *a*(0), *b*(0), *c*(0), *d*(0), *e*(0) with an increment of *MTU*(*M*_2_) = 42, and *a*(1), *b*(1), *c*(1), *d*(1), *e*(1) with an increment of *MTU*(*M*_1_) = 48. The resulting MTWU table is shown in Table 11.

**Table 11:** MTWU values after scanning $\langle 2,T_{2}\rangle$.

X Span	*a*	*b*	*c*	*d*	*e*	*f*	*g*
0	42	85	85	85	42	0	43
1	43	43	43	43	43	0	0
2	0	0	0	0	0	0	0

In Step 3, we follow the same process as before, with a different value of *u* and *max*. Specifically, with *u* = 3 and *max* = 2, the items in the transaction *T*_3_ will generate extension-items with *span* = 0, *span* = 1, and *span* = 2. We need to update the values in the MTWU table accordingly. The updates required are:


$$\begin{array}{c} \begin{array}{cc} & a(0),c(0),d(0)\text{ with }MTU(M_{3})=42,\\ & a(1),c(1),d(1)\text{ with }MTU(M_{2})=42,\\ & a(2),c(2),d(2)\text{ with }MTU(M_{1})=43. \end{array} \end{array}$$

After updating, the MTWU table is shown in Table 12:

**Table 12:** MTWU values after scanning $\langle 3,T_{3}\rangle$.

X Span	*a*	*b*	*c*	*d*	*e*	*f*	*g*
0	84	85	127	127	42	0	43
1	85	43	85	85	43	0	0
2	43	0	43	0	43	0	0

Please refer to Table 6 for the results obtained from continuing work on the remaining transactions.

**Building a Total Order for Extension-Items**

Since the proposed algorithm does not construct a mega-transaction database, it directly processes the original transactions containing the initial items. Before performing the sorting procedure, the algorithm eliminates items whose extension MTWU values are lower than the user-defined threshold *minUtil*. For example, item *g* is removed because its MTWU does not satisfy the threshold condition. The remaining items are then sorted in ascending order according to their MTWU values at *span* = 0. Consequently, the items are arranged in the following order:


$$\begin{array}{c} f≻e≻a≻b≻d≻c. \end{array}$$

Next, a global total order is established based on the following principle:

- Extension-items with smaller spans are considered larger.
- If two extension-items have the same span, they are ordered in ascending order according to the MTWU value of their corresponding item at *span* = 0.

Based on this strategy, in the running example, the total order ≻ is defined as:


$$\begin{array}{c} f(0succe(0)≻a(0)≻b(0)≻d(0)≻c(0)≻f(1)≻e(1)≻a(1)≻b(1)≻d(1)≻c(1)≻f(2)≻e(2)≻a(2)≻b(2)≻d(2)≻c(2)\text{.} \end{array}$$

After establishing the global order, the algorithm removes unpromising extension-items from each transaction and sorts the remaining items according to ≻. Empty transactions are retained in the database. The resulting database is presented in Table 13.

**Table 13:** Database *D* after updating by ≻.

Tid	Transaction
1	*b*(1), *d*(2), *c*(2)
2	*e*(2), *a*(4), *b*(1), *d*(1), *c*(3)
3	*a*(4), *d*(1), *c*(2)
4	*f*(1), *e*(1), *c*(2)
5	*e*(2), *a*(5), *b*(2), *d*(1)
6	*f*(2), *a*(3), *b*(4), *c*(1)

### Complexity analysis

We analyze the complexity of the algorithms in terms of time and space in this section.

**Baseline Algorithm:**

Based on the published complexity of the iEFIM algorithm, which is O(l×n×w), where *l* denotes the number of itemsets in the search space, *n* is the number of transactions, and *w* is the average transaction length in the transaction database *D*.

However, in the problem of mining inter-transaction high-utility itemsets, each mega-transaction is constructed by combining up to maxSpan ℒ consecutive transactions. As a result, the average transaction length is no longer *w*, but is extended to: $w^{\prime }=ℒ\times w$.

Therefore, the complexity of iEFIM in this context is $O(l\times n\times w^{\prime })$.

Besides the main mining process, the algorithm also needs to perform an additional step of normalizing the threshold-satisfying candidates to check whether they duplicate previously discovered ITHUIs.

The cost of this step includes the candidate normalization time, $O(w^{\prime })$, and the time required to compare the candidate with the mined ITHUI sets, $O(w^{\prime }\times size)$, where *size* is the number of ITHUIs found and *size* < *l*.

Based on these components, the complexity of iEFIM for the ITHUI mining task can be written as:


$$\begin{array}{c} O(l\times n\times w^{\prime }+w^{\prime }\times size)=O(l\times n\times w^{\prime }) \end{array}$$

In other words, the extra work for normalization and duplicate checking increases the running time in practice, but it does not change the overall complexity, which remains $O(l\times n\times w^{\prime })$.

**Improved Algorithm:**

In the improved algorithm, candidates that satisfy the *minUtil* threshold do not need to be checked against previously mined ITHUIs for duplication. Instead, the algorithm only normalizes each candidate and verifies whether its first item has *span* = 0. As a result, the cost of duplicate checking is eliminated.

Therefore, the complexity of the improved algorithm can be expressed as:


$$\begin{array}{c} O(l\times n\times w^{\prime }+w^{\prime })=O(l\times n\times w^{\prime }) \end{array}$$

Although the improved and baseline algorithms have the same complexity order, $O(l\times n\times w^{\prime })$, their actual running times may differ. The baseline algorithm needs extra time to check whether a newly generated ITHUI has already been found. In contrast, the improved algorithm skips this step. As a result, the improvement is more effective when many ITHUIs are produced during mining.

**Space Complexity:**

For the baseline algorithm, the space complexity is $O(ℒ\times |I|+l\times n\times w^{\prime }\times k)+O(size)$, where *k* denotes the storage space required for Psets, and *size* represents the number of mined ITHUIs. Since *size* is smaller than *l*, the general space comp*l*exity of the baseline algorithm can be simplified as:


$$\begin{array}{c} O(ℒ\times |I|+l\times n\times w^{\prime }\times k) \end{array}$$

Since the improved algorithm does not perform duplicate checking, it does not need to keep all previously discovered ITHUIs for comparison. Thus, its space complexity is:


$$\begin{array}{c} O(ℒ\times |I|+l\times n\times w^{\prime }\times k) \end{array}$$

In theory, the baseline and improved algorithms have the same space complexity. However, this does not mean that they use the same amount of memory in practice. For dense databases, or when the *minUtil* threshold is set to a low value, the number of discovered ITHUIs can be very large. In this case, the baseline algorithm needs extra memory to store the mined ITHUIs for duplicate checking. This may lead to a considerable increase in memory usage. By contrast, the improved algorithm does not require this additional storage, so it can save memory when many ITHUIs are generated during mining.

## Experimental results

We evaluated the performance of three algorithms: ITHUIM, ITHUIMZ, and BitITHUIM. All experiments were conducted in a Java environment on a Dell Latitude 7490 laptop equipped with an Intel Core i7-8650U 1.9 GHz processor, 32 GB of RAM, and running the Windows 10 operating system. The experimental datasets were obtained from the SPMF library [35]. The detailed characteristics of the datasets are summarized in Table 14. We compare and analyze the performance of the three algorithms with respect to three main criteria: execution time, and memory consumption.

**Table 14:** Dataset properties.

Dataset	*n*	|*I*|	Avg Length	Density
Retail	88,162	16,470	10.3	0.0600
Kosarak	990,002	41,270	8.1	0.0196

With the selected databases, the experiments are executed with *maxSpan* values ranging from 2 to 5. To ensure accuracy, each experiment was run at least 10 times, and the results represent the average of these executions. The experimental results show that the algorithm has exploited the ITHUI sets on the test databases.

First, this research compares the experimental results of the algorithms on runtimes and memory consumption, including: ITHUIM, BitITHUIM, and ITHUIMZ.

As shown in Table 15, both the BitITHUIM and ITHUIMZ algorithms outperform the ITHUIM algorithm in terms of runtime and memory efficiency. The main reason is that ITHUIMZ encodes extension-items into a single integer representation, which enables faster computation and more efficient processing. In contrast, in ITHUIM, each extension-item is stored using two separate components, namely the item and its span, resulting in higher memory consumption and longer processing time. The BitITHUIM algorithm operates on a bit-based structure; therefore, additional processing time is required to manipulate the bit representation in order to extract span and item information. Consequently, its execution time is slightly longer than that of ITHUIMZ. Furthermore, as the *minUtil* threshold increases, the number of generated candidates decreases. This reduction leads to a significant decrease in memory usage for ITHUIMZ, allowing it to further outperform BitITHUIM.

**Table 15:** Performance on Kosarak with *maxSpan* = 2 and 3.

*minUtil*	*maxSpan*	Runtimes (ms) (Mean ± SD)			Memory (MB) (Mean ± SD)		
		ITHUIM	BitITHUIM	ITHUIMZ	ITHUIM	BitITHUIM	ITHUIMZ
1,000,000	2	40,288 ± 956	34,263 ± 883	28,497 ± 1,132	4,407 ± 58	1,950 ± 50	3,629 ± 298
1,500,000		18,857 ± 619	14,359 ± 705	13,432 ± 684	2,860 ± 26	1,754 ± 42	2,244 ± 232
2,000,000		12,806 ± 673	9,859 ± 300	9,645 ± 532	2,158 ± 23	1,504 ± 46	1,430 ± 217
2,500,000		9,683 ± 426	7,418 ± 600	7,148 ± 413	1,976 ± 26	1,327 ± 37	1,210 ± 383
1,000,000	3	74,010 ± 1,632	66,646 ± 1,875	62,717 ± 2,311	7,127 ± 113	8,316 ± 145	5,813 ± 245
1,500,000		45,080 ± 1,230	36,337 ± 1,243	34,910 ± 1,105	4,765 ± 108	3,829 ± 88	3,869 ± 126
2,000,000		29,502 ± 859	25,114 ± 713	23,487 ± 825	4,027 ± 103	3,074 ± 75	2,963 ± 112
2,500,000		22,996 ± 654	18,037 ± 431	17,937 ± 358	3,336 ± 96	2,548 ± 58	2,374 ± 96

The study was also conducted to evaluate the algorithms when the *maxSpan* parameter varies under a fixed *minUtil* threshold. The results presented in Table 16 indicate that both the bitwise storage structure and the integer encoding of extended items continue to provide superior performance in terms of execution time and memory consumption.

**Table 16:** Performance on Kosarak on different *maxSpan.*

*maxSpan*	*minUtil*	Runtimes (ms) (Mean ± SD)			Memory (MB) (Mean ± SD)		
		ITHUIM	BitITHUIM	ITHUIMZ	ITHUIM	BitITHUIM	ITHUIMZ
2	1,500,000	18,857 ± 619	14,359 ± 705	13,432 ± 684	2,860 ± 26	1,754 ± 42	2,244 ± 232
3		45,080 ± 1,230	36,337 ± 1,243	34,910 ± 1,105	4,765 ± 108	3,829 ± 88	3,869 ± 126
4		98,528 ± 2,503	81,210 ± 2,134	86,632 ± 2,067	7,031 ± 215	7,180 ± 253	7,377 ± 240
5		239,065 ± 5,874	207,910 ± 5,145	208,274 ± 5,923	9,083 ± 373	9,798 ± 246	12,491 ± 312
2	2,000,000	12,806 ± 673	9,859 ± 300	9,645 ± 532	2,158 ± 23	1,504 ± 46	1,430 ± 217
3		29,502 ± 859	25,114 ± 713	23,487 ± 825	4,027 ± 103	3,074 ± 75	2,963 ± 112
4		72,474 ± 1,854	58,263 ± 1,488	56,635 ± 1,632	6,587 ± 191	5,545 ± 187	5,103 ± 181
5		170,077 ± 4,952	142,419 ± 4,914	130,904 ± 4,754	10,118 ± 455	11,048 ± 410	7,893 ± 317

**Table 18:** Performance on Retail with *maxSpan* = 4 and 5.

*minUtil*	*maxSpan*	Runtimes (ms) (Mean ± SD)			Memory (MB) (Mean ± SD)		
		ITHUIM	BitITHUIM	ITHUIMZ	ITHUIM	BitITHUIM	ITHUIMZ
200,000	4	48,334 ± 4,374	49,582 ± 4,312	47,263 ± 4,221	3,833 ± 253	3,586 ± 246	3,575 ± 231
600,000		11,541 ± 1,153	11,171 ± 1,031	10,809 ± 921	1,902 ± 154	1,916 ± 146	1,700 ± 132
800,000		8,420 ± 759	7,155 ± 712	7,115 ± 683	1,626 ± 96	1,175 ± 84	1,164 ± 87
1,000,000		6,487 ± 519	5,735 ± 415	5,269 ± 384	1,618 ± 94	815 ± 65	1,103 ± 84
200,000	5	134,225 ± 12,548	138,979 ± 11,453	140,662 ± 13,429	5,302 ± 434	4,846 ± 389	4,977 ± 403
600,000		49,171 ± 3,875	49,084 ± 4,123	45,751 ± 3,954	3,989 ± 326	4,101 ± 340	3,761 ± 304
800,000		28,959 ± 1,564	26,961 ± 1,478	27,502 ± 1,435	3,595 ± 276	3,334 ± 256	3,166 ± 240
1,000,000		12,755 ± 821	11,896 ± 769	11,251 ± 805	2,457 ± 219	2,136 ± 198	2,068 ± 176

Similar results are also presented in Tables 17-19, which report the experimental results on the Retail dataset.

**Table 17:** Performance on Retail with *maxSpan* = 2 and 3.

*minUtil*	*maxSpan*	Runtimes (ms) (Mean ± SD)			Memory (MB) (Mean ± SD)		
		ITHUIM	BitITHUIM	ITHUIMZ	ITHUIM	BitITHUIM	ITHUIMZ
200,000	2	5,863 ± 215	5,420 ± 278	4,930 ± 204	753 ± 57	890 ± 77	842 ± 70
600,000		2,086 ± 95	1,991 ± 134	1,849 ± 117	528 ± 43	429 ± 65	413 ± 46
800,000		1,831 ± 82	1,442 ± 75	1,389 ± 61	309 ± 41	297 ± 35	281 ± 33
1,000,000		1,360 ± 56	1,204 ± 47	1,382 ± 52	271 ± 37	263 ± 42	247 ± 33
200,000	3	18,392 ± 625	16,933 ± 588	16,070 ± 583	2,461 ± 135	2,609 ± 132	2,287 ± 129
600,000		5,306 ± 273	4,830 ± 191	4,662 ± 219	1,083 ± 74	747 ± 59	838 ± 67
800,000		4,075 ± 231	3,365 ± 152	3,412 ± 153	942 ± 69	629 ± 51	663 ± 51
1,000,000		3,069 ± 186	2,540 ± 123	2,662 ± 103	806 ± 52	481 ± 37	468 ± 39

**Table 19:** Performance on Retail on different *maxSpan.*

*maxSpan*	*minUtil*	Runtimes (ms) (Mean ± SD)			Memory (MB) Mean ± SD)		
		ITHUIM	BitITHUIM	ITHUIMZ	ITHUIM	BitITHUIM	ITHUIMZ
2	200,000	5,863 ± 215	5,420 ± 278	4,930 ± 204	753 ± 57	890 ± 77	842 ± 70
3		18,392 ± 625	16,933 ± 588	16,070 ± 583	2,461 ± 135	2,609 ± 132	2,287 ± 129
4		48,334 ± 4,374	49,582 ± 4,312	47,263 ± 4,221	3,833 ± 253	3,586 ± 246	3,575 ± 231
5		134,225 ± 12,548	138,979 ± 11,453	140,662 ± 13,429	5,302 ± 434	4,846 ± 389	4,977 ± 403
2	600,000	2,086 ± 95	1,991 ± 134	1,849 ± 117	528 ± 43	429 ± 65	413 ± 46
3		5,306 ± 273	4,830 ± 191	4,662 ± 219	1,083 ± 74	747 ± 59	838 ± 67
4		11,541 ± 1,153	11,171 ± 1,031	10,809 ± 921	1,902 ± 154	1,916 ± 146	1,700 ± 132
5		49,171 ± 3,875	49,084 ± 4,123	45,751 ± 3,954	3,989 ± 326	4,101 ± 340	3,761 ± 304

Next, we compare the performance of the improved strategy against the baseline ITHUIM algorithm.

Fig 1 illustrates the difference in runtimes, memory consumption, and the number of generated candidates between two cases of the ITHUIM algorithm: with Improvement 1 applied (Ver2) and without Improvement 1 (Ver1). By pruning candidates that do not start with *span* = 0 during the ITHUI mining process, the algorithm in Ver2 eliminates a large number of candidates generated from items whose spans are different from 0. As a result, the number of explored candidates is significantly reduced, allowing the algorithm to operate more efficiently in terms of both memory consumption and execution time.

**Fig 1:**
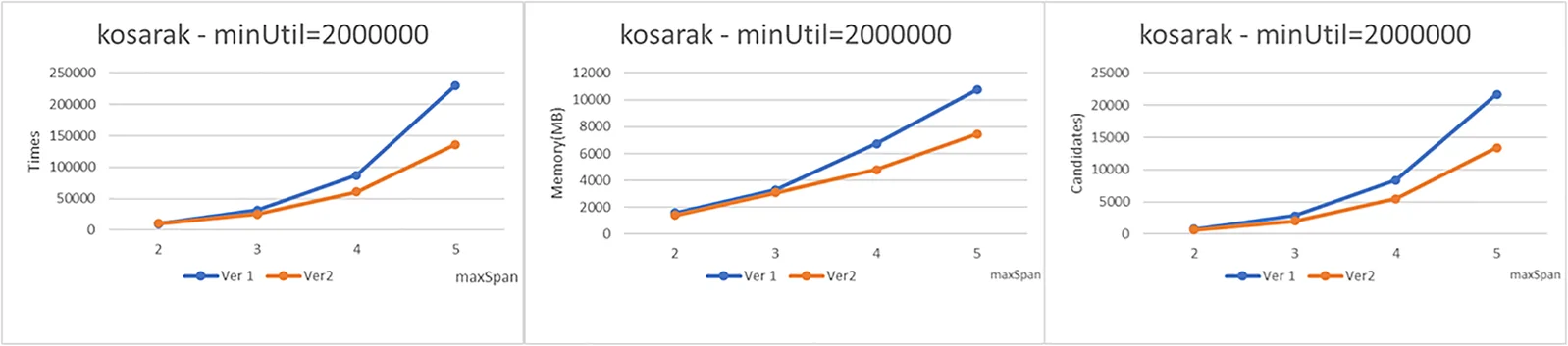
Runtime, memory and candidates performance on Kosarak with Improvements.

Moreover, ignoring items with spans other than 0 prevents the generation of duplicate ITHUIs, as proven in the previous section. Consequently, the algorithm does not need to spend additional time determining the centers of ITHUIs during the mining process. This reduction in redundant computations is one of the key factors contributing to the improved and storage efficiency of the proposed approach.

Tables 20 and 21 reveal several important patterns regarding the effectiveness of the proposed optimizations.

**Table 20:** Average improvement ratios of BitITHUIM and ITHUIMZ over the baseline ITHUIM on Kosarak (*Positive values indicate improvement; negative values indicate overhead*).

*maxSpan*	Runtimes		Memory		*minUtil*	Runtimes		Memory	
	BitHUIM	ITHUIMZ	BitHUIM	ITHUIMZ		BitHUIM	ITHUIMZ	BitHUIM	ITHUIMZ
2	21.3	27.2	39.4	27.9	1,500,000	18.5	19.1	12.1	−0.5
3	16.5	20.1	12.6	23.1	2,000,000	18.4	22.5	15.2	26.2
Average	18.9	23.7	26.0	25.5	Average	18.5	20.8	13.7	12.4

**Table 21:** Average improvement ratios of BitITHUIM and ITHUIMZ over the baseline ITHUIM on Retail (*Positive values indicate improvement; negative values indicate overhead*).

*maxSpan*	Runtimes		Memory		*minUtil*	Runtimes		Memory	
	BitHUIM	ITHUIMZ	BitHUIM	ITHUIMZ		BitHUIM	ITHUIMZ	BitHUIM	ITHUIMZ
2	11.2	12.5	1.9	7.0	200,000	2.4	6.5	−2.3	2.0
3	12.9	13.6	24.6	25.3	600,000	4.3	9.2	24.9	15.2
4	6.8	10.7	20.8	19.4	800,000	15.1	15.2	18.0	19.8
5	2.6	4.8	6.6	9.9	1,000,000	11.8	14.6	26.5	24.6
Average	8.4	10.4	13.5	15.4	Average	8.4	10.4	13.4	15.4

**Dataset-level comparison.** Both BitITHUIM and ITHUIMZ achieve substantially higher runtime improvements on Kosarak (18.9% and 23.7% on average, respectively) than on Retail (8.4% and 10.4%). This difference is attributable to the larger scale of Kosarak (990,002 transactions and 41,270 distinct items vs. 88,162 transactions and 16,470 items in Retail): larger databases generate more mega-transactions and longer utility-lists, amplifying the per-operation savings provided by compact integer encoding and bitmap-based intersection. In contrast, Retail’s smaller scale provides less computational overhead for the optimizations to reduce.

**Effect of maxSpan.** On the Retail dataset, as *maxSpan* increases from 2 to 5, the average runtime improvement of BitITHUIM decreases from 11.2% to 2.6%, and that of ITHUIMZ from 12.5% to 4.8%. This decline occurs because larger *maxSpan* values produce mega-transactions with significantly more extension-items, exponentially expanding the search space. Under such conditions, the dominant cost shifts from utility-list operations, where the proposed optimizations are most effective, to the sheer volume of candidate itemsets to be explored. On Kosarak, the runtime improvement remains higher and more stable across *maxSpan* values (16.5% – 21.3% for BitITHUIM; 20.1% – 27.2% for ITHUIMZ), confirming that the optimizations scale more favorably on larger, sparser datasets.

**Effect of minimum utility thresholds (Retail).** Memory improvement increases monotonically with *minUtil* for both algorithms: BitITHUIM improves from −2.3% at *minUtil* = 200*K* to 26.5% at *minUtil* = 1,000*K*, while ITHUIMZ improves from 2.0% to 24.6% over the same range. Higher minimum utility thresholds enable more aggressive TWU-based pruning, reducing the number of candidates stored in memory and allowing the compact data structures to operate on a smaller, more manageable search space. At *minUtil* = 200*K*, the search space is very large and the overhead of maintaining the bitmap representation slightly exceeds the savings, resulting in a −2.3% overhead for BitITHUIM. Notably, even at this most challenging threshold, ITHUIMZ still achieves a positive memory improvement (2.0%), demonstrating that the compact encoding introduces less structural overhead than the bitmap approach. Runtime improvement peaks at *minUtil* = 800*K* (approximately 15% for both algorithms) and slightly decreases at *minUtil* = 1,000*K*, because the search space at the highest threshold is already very small, limiting further percentage gains due to fixed overhead costs such as I/O and initialization.

**Memory trade-offs.** BitITHUIM achieves its best memory reduction at small *maxSpan* on Kosarak (39.4% at *maxSpan* ℒ=2) due to the compact bitmap representation. However, as *maxSpan* increases, the number of extension-items grows and the bitmap overhead rises, reducing the memory advantage (12.6% at *maxSpan* ℒ=3). Similarly, ITHUIMZ exhibits a slight memory overhead (−0.5%) on Kosarak at *minUtil* = 1.5*M*, where the very large candidate set causes the structural cost of the integer-encoded utility-lists to marginally exceed the per-item savings. At *minUtil* = 2.0*M*, however, the pruned candidate set is sufficiently smaller for the encoding savings to dominate, yielding a 26.2% memory improvement. These results indicate that neither optimization uniformly dominates across all parameter settings; the choice between BitITHUIM and ITHUIMZ should be guided by the expected *maxSpan* and dataset characteristics.

**Comparison between ITHUIMZ and BitITHUIM.** Table 22 summarizes the average improvement ratios across both datasets and parameter dimensions. Across all configurations, ITHUIMZ consistently achieves higher runtime improvements than BitITHUIM (10.4% vs. 8.4% on Retail; 22.2% vs. 18.7% on Kosarak). For memory, the comparison is more nuanced: on Retail, ITHUIMZ is generally more effective (15.4% – 17.2% vs. 13.4% – 13.5%), while on Kosarak the two approaches yield comparable savings (19.2% vs. 19.8%). These results indicate that the compact integer encoding strategy offers a more robust and broadly effective optimization than the bitmap-based approach, particularly in terms of computational speed. Notably, at minUtil≥800K on Retail, none of the 16 metric values (across 4 *maxSpan* settings × 2 metrics) is negative for ITHUIMZ, confirming that the proposed optimizations are consistently effective under practical mining thresholds.

**Table 22:** Average improvement ratios (%) of BitITHUIM and ITHUIMZ.

Dataset	Dimension	Runtimes		Memory	
		ITHUIMZ	BitITHUIM	ITHUIMZ	BitITHUIM
Retail	by *maxSpan*	10.4	8.4	17.2	13.5
Retail	by *minUtil*	10.4	8.4	15.4	13.4
Kosarak	by *maxSpan*	23.6	18.9	25.5	26.0
Kosarak	by *minUtil*	20.8	18.4	12.8	13.6

**Limitations on dense databases.** One limitation of the proposed algorithms is their reduced efficiency on dense databases, where transactions contain a high proportion of the total item set. In such databases, each mega-transaction, constructed by concatenating up to ℒ consecutive original transactions, can contain a very large number of extension-items, approaching ℒ×|I| in the worst case, where |*I*| is the number of distinct items per transaction. This causes the search space to grow exponentially with *maxSpan*, as the number of candidate itemsets derived from each mega-transaction increases combinatorially. Consequently, the utility-list construction and intersection operations, which are the core computational steps of all three proposed variants, become significantly more expensive, and the pruning strategies (TWU-based and span-based) lose their effectiveness because utility upper bounds remain high in dense settings. Our experimental results on Retail with *maxSpan* ℒ=5 already illustrate this trend: the runtime of ITHUIM increases by approximately 22.9 times compared to *maxSpan* ℒ=2 (from 5,863 ms to 134,225 ms), and the improvement ratios of BitITHUIM and ITHUIMZ diminish to only 2.6% and 4.8%, respectively. Developing more effective pruning strategies for dense inter-transaction databases remains an important direction for future research.

## Conclusion

The study proposed the problem and concepts related to mining inter-transaction high utility itemsets (ITHUIs) and established the related definitions and properties. On this basis, the study also applied the iEFIM algorithm to efficiently mine ITHUIs. Additionally, we proposed two methods for storing extension-items to reduce memory consumption during the ITHUIs mining process. At the same time, processing only items with a span equal to 0 in the first stage helped the algorithm significantly reduce the number of candidates and shorten the execution time. However, when combining consecutive transactions, the number of extension-items increases substantially. This is the reason why the number of candidates increases exponentially and causes difficulties when executing on dense databases.

In the future, we will focus on avoiding the transformation process to a mega database to reduce storage space. Furthermore, our research will explore the specific characteristics of the mega-transaction database to further prune the search space.
